# Supplementary material for: VueGen: automating the generation of scientific reports
Source: Bioinform Adv. 2025 Jun 24;5(1):vbaf149. doi: 10.1093/bioadv/vbaf149 (PMC12233086; doi:10.1093/bioadv/vbaf149)
Supplement: vbaf149_Supplementary_Data [file vbaf149_supplementary_data.docx]

| **Supplementary Information**  **VueGen: Automating the generation of scientific reports**  Sebastian Ayala-Ruano^1^, Henry Webel^1^, Alberto Santos^1,*^  ^1^Multiomics Network Analytics Group, Novo Nordisk Foundation Center for Biosustainability, Technical University of Denmark, Building 220 Søltofts Plads, 2800 Kongens, Lyngby, Denmark  *Corresponding author. E-mail: albsad@dtu.dk |
| --- |


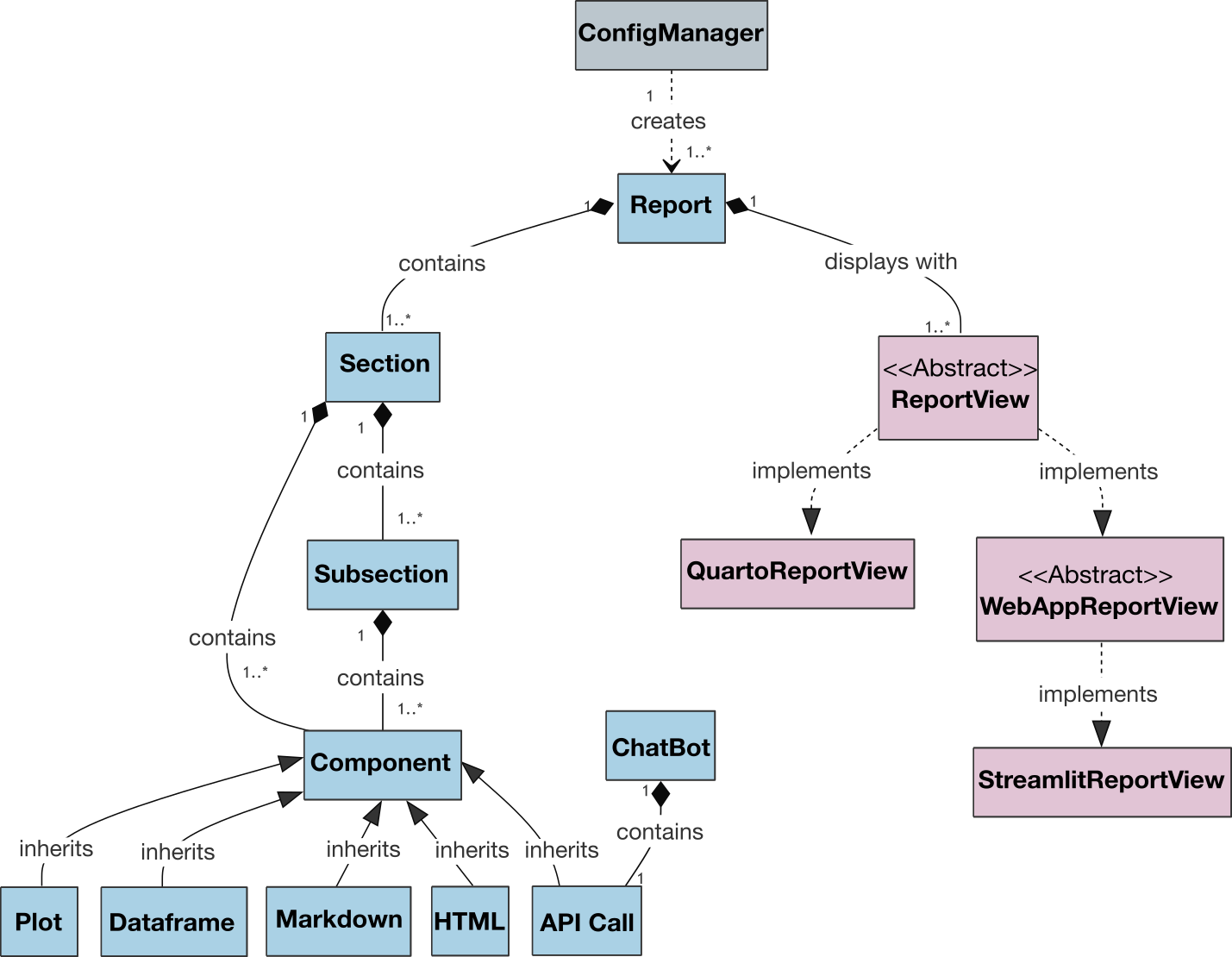


**Figure S1. VueGen class diagram (v0.4.0).** Boxes represent classes, and arrows show their relationships. Colors indicate functionality: gray for classes managing input files and metadata, blue for those defining the report structure, and red for classes generating reports.

**Table S1. Comparison of VueGen with existing reporting tools in terms of supported formats, usability, interactivity, extensibility, and intended purpose.** The table highlights VueGen’s unique strengths in automating report generation, integrating heterogeneous content, and offering flexibility across user expertise levels.

| **Feature** | **VueGen** | **Quarto** | **Streamlit** | **MultiQC** |
| --- | --- | --- | --- | --- |
| **Report Formats** | HTML, PDF, DOCX, ODT, PPTX, Reveal.js, Jupyter notebook, and web application | HTML, PDF, DOCX, ODT, PPTX, Reveal.js, and Jupyter notebook | Web application | HTML, PDF |
| **Coding Requirements** | No coding for basic reports; Python, YAML, and Markdown for customization | Markdown and YAML for basic reports; Python, R, JS, or HTML for advanced customization | Requires Python for creating web applications | No coding for basic reports; YAML, JS, or HTML for custom reports |
| **Installation Options** | - Python package - Docker image - nf-core module - Desktop application | - Python package - Docker image | - Python package - Docker image | - Python package - Docker image - nf-core module |
| **Report Purpose** | Scientific reports, extendable to other fields | Scientific reports, extendable to other fields | Web applications and dashboards | Quality control reports for bioinformatics analyses |
| **Data Integration** | Automated: Integrates plots, tables, networks, HTML, and markdown components from folder structure or config file | Manual: Users must write code or Quarto directives to load and render each component | Manual: Users must write Python code to load and display each element | Semi-automated: Aggregates data from module outputs, but requires configuration and formatting for custom content |
| **Interactivity and Dynamic Content** | Fully interactive and dynamic: supports Plotly, Altair, Pyvis, tables, and dynamic components like API calls for real-time updates | Interactive: supports Plotly, Altair, and HTML widgets natively; dynamic components achievable via Shiny implementation, Observable JS, or custom JS integrations | Fully interactive and dynamic: provides built-in support for interactive widgets, dynamic components, and custom logic using Python code | Interactive: built-in interactivity for plots (Plotly) and tables (custom JavaScript); limited customization options |
| **Report Extensibility** | High: generated reports can be extended by editing the underlying Python or QMD files, allowing full customization | High: designed for manual editing and scripting; supports code from multiple languages, HTML snippets, and custom plugins | High: full extensibility via Python scripting; ideal for building custom logic and layouts | Intermediate: supports custom content and plugins, but limited customization options; only one custom plot per section allowed; extending reports requires familiarity with internal structures and scripting |
| **Report Structure** | Supports both single-document (e.g., HTML, PDF, DOCX) and multi-page web apps; Streamlit reports use sidebar navigation with one page per subsection; multiple components per section allowed | Linear layout with optional sidebar navigation; content rendered in a continuous scroll | Flexible layout (single or multi-page); navigation via sidebar or tabs, depending on implementation | Linear layout with sidebar navigation; content rendered in a continuous scroll; only one custom plot per section allowed |
